# Night Nurses' Meals

**Published:** 1919-07-26

**Authors:** 


### NIGHT NURSES' MEALS.

In the Report recently issued by the Salaries Committee appointed by the Council of the College of Nursing, on the general conditions of employment of nurses, many interesting facts were disclosed relating to the inside affairs that control and govern the personal life of the nurse in institutions. It elicited the by no means uncommon experience that there are night nurses working an average of eighty-four hours a week. Allied to this is the question of the food supplied to night nurses to enable them to endure the strain of night work and carry out their duties in a fitting manner. Night nurses ought to be supplied with as full a complement of meals as is provided to the day staff, and to have a hot meal in the dining-room, properly served and attended. Unfortunately, it is well known that this arrangement is not universal, nor is it in force even in some of our leading institutions. Those institutions whose night staff sit down to a hot meal out of the wards between the hours of 12 midnight and 2 A.M. are very few in number. The first meal, termed breakfast, taken usually at 7.30 P.M., consists of bacon, ham, or fish, and the dinner on coming off duty at 9 A.M., of meat, vegetables, and pudding. Apart from this, the nightly allowance for two people usually consists of half a loaf, a pat of butter, a small portion of jam sometimes, and an egg apiece. Tea and sugar are rationed out weekly, and, since the present ruling of high prices, even the egg has disappeared. In some institutions an allowance of money is given in lieu of rations. This is a system that has not proved at all satisfactory, for many nurses buy too little and of poor quality under some mistaken notion of being economical. Too often the night staff receive only the after-thoughts of consideration from the commissariat department. In large institutions possessing every facility and an up-to-date steam or electric cooking apparatus, it is difficult to understand why this should be. There are greater difficulties where the equipment of the kitchen is still dependent upon old-fashioned coal-fire machinery; yet a little expenditure of thought and administrative skill, a little extra manipulation and management would easily provide one good meal during the night. Moreover, it should take a special place in the curriculum of housekeeping, and be enforced by all hospital managers and inspectors. Neglect of such an important matter as the provision of properly served meals during the night is not to be condoned. That nurses require the stimulus of good wholesome food, most especially during the closed-in, devitalising hours of the night, should be fully acknowledged, and become the charge of all who have their material welfare at heart.

